# Cardiac Protective Role of Heat Shock Protein 27 in the Stress Induced by Drugs of Abuse

**DOI:** 10.3390/ijms21103623

**Published:** 2020-05-21

**Authors:** Elena Martínez-Laorden, Javier Navarro-Zaragoza, María Victoria Milanés, María Luisa Laorden, Pilar Almela

**Affiliations:** Department of Pharmacology, Faculty of Medicine, University of Murcia, 30100 Murcia, Spain; elena.m.l@um.es (E.M.-L.); milanes@um.es (M.V.M.); laorden@um.es (M.L.L.); palmela@um.es (P.A.)

**Keywords:** heat shock protein 27, morphine withdrawal, stress, heart

## Abstract

Heat shock proteins (HSP) are induced after different stress situations. Some of these proteins, particularly HSP-27, function as markers to indicate cellular stress or damage and protect the heart during addictive processes. Morphine withdrawal induces an enhancement of sympathetic activity in parallel with an increased HSP-27 expression and phosphorylation, indicating a severe situation of stress. HSP-27 can interact with different intracellular signaling pathways. Propranolol and SL-327 were able to antagonize the activation of hypothalamic-pituitary adrenal (HPA) axis and the phosphorylation of HSP-27 observed during morphine withdrawal. Therefore, β-adrenergic receptors and the extracellular signal-regulated kinase (ERK) pathway would be involved in HPA axis activity, and consequently, in HSP-27 activation. Finally, selective blockade of corticotrophin releasing factor (CRF)-1 receptor and the genetic deletion of CRF1 receptors antagonize cardiac adaptive changes. These changes are increased noradrenaline (NA) turnover, HPA axis activation and decreased HSP-27 expression and phosphorylation. This suggests a link between the HPA axis and HSP-27. On the other hand, morphine withdrawal increases µ-calpain expression, which in turn degrades cardiac troponin T (cTnT). This fact, together with a co-localization between cTnT and HSP-27, suggests that this chaperone avoids the degradation of cTnT by µ-calpain, correcting the cardiac contractility abnormalities observed during addictive processes. The aim of our research is to review the possible role of HSP-27 in the cardiac changes observed during morphine withdrawal and to understand the mechanisms implicated in its cardiac protective functions.

## 1. Introduction

Stress proteins are classified into five families based on their amino acid sequence and molecular weight: 1) low molecular weight (15–30 kDa), 2) 60 kDa, 3) 70 kDa, 4) 90 kDa, and 5) high molecular weight (100–110 kDa) [1]. As in the case of other heat shock proteins (HSP), low molecular weight thermal shock proteins provide thermotolerance to cells and participate in other processes, such as cytoskeleton stabilization and apoptosis regulation [2]. The synthesis of HSP, which is expressed in different tissues, arises as a consequence of a sudden increase in temperature. Several studies have revealed an increased HSP expression not only after hyperthermia but also as a defense mechanism against various physical and chemical agents that induce cellular stress. Therefore, HSP are also known as stress proteins.

**Figure 1:**
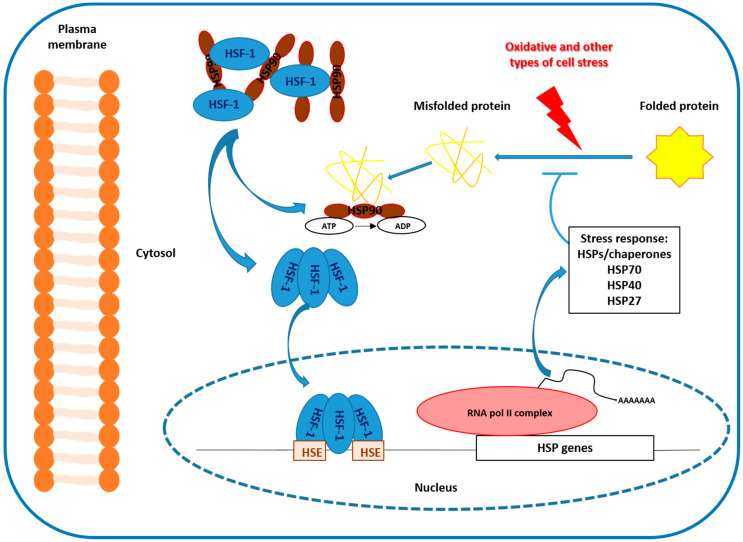
Under non-stress conditions, heat shock transcription factor (HSF) is located in the cell cytoplasm as a monomer and lacks the ability to bind to heat shock elements (HSE) situated in the promoters of heat shock protein (HSP) genes. However, under a stimulus such as an increase in temperature or exposure to a stressor, HSF is phosphorylated by mitogen-activated protein kinase and converted into a DNA-binding trimer. This allows the transcription of HSP genes that directly increases the synthesis of HSP proteins to sufficiently high levels, repairing damaged proteins. Finally, these chaperones are re-associated again with HSF, recovering their inactive state.

Stress proteins expression is regulated at the transcriptional level [1,3]. The increase in transcription requires the binding of a heat shock transcription factor to a sequence located in the promoter region of all heat-inducible genes [4]. Under normal conditions, heat shock transcription factor is located in the cell cytoplasm in a monomeric form remaining dormant without the ability to bind to DNA. However, after a stimulus, like an increase in temperature or exposure to a stressful agent, heat shock transcription factor is phosphorylated by action of the mitogen activated protein kinase, which forms trimers. This trimerization is essential for its function, providing the necessary affinity to translocate to the nucleus and joining to the promoter zone of the gene that encodes the different HSP [5,6]. Consequently, HSP genes transcription can be carried out, increasing the synthesis of HSP proteins to high levels, which allow the repairment of the damaged proteins. Once their mission is accomplished, these chaperones are again associated with the heat shock transcription factor, restoring its inactive state (Figure 1).

Most of stress proteins are found constitutively in different tissues, participating in various cellular processes, while the rest are normally located at low concentrations or at almost undetectable levels, being rapidly induced by stress [6]. Almost all HSP are capable of transiently bind to a wide variety of cellular proteins. HSP can act as molecular chaperones and to intervene in the repairment of denatured proteins after damage and in the balance between synthesis, assembly, and degradation [7].

It is known that HSP participate in a series of biological processes, from the cell cycle to the cell differentiation, from adaptation to stressful conditions, in apoptosis and even in the transformation of a cell into a malignant state. Moreover, HSP malfunction has been implicated in different cardiac alteration and in the development of neurological diseases. However, its protective role in stress situations associated to addiction processes is not well known. Therefore, in this review, we summarize the latest news and views, including our own studies, that describe and discuss the cardiac protective role of HSP-27 in the stress induced by drugs of abuse.

## 2. Heat Shock Protein 27 (HSP-27) and Stress

In humans, HSP-27 is one of the most important low molecular weight HSP. It is constitutively located in almost all cell types and tissues, but mainly expressed in cardiac tissue [8]. HSP-27 post-translational modification is carried out by phosphorylation at three serine residues (Ser 15, Ser 78, and Ser 82). This phosphorylation is mainly carried out by mitogen activated extracellular signals, although other protein kinases such as protein kinase D, in cell lines and pancreas, and protein kinase C, can also phosphorylate HSP-27, primarily in the residue Ser 82 [3].

The essential proteins involved in the mechanism of cardiac contraction are actin, myosin, and troponins (Tn). In 1954, Huxley and Hanson [9] proposed the “sliding filament theory”, which states that muscle contraction is the result of the relative slippage of myosin filaments (thick filaments) and actin filaments (thin filaments). Tn is activated in the presence of Ca^2+^ to expose the actin and myosin binding site. Troponin is a complex formed by three proteins (TnT, TnI, and TnC), each one with a specific function: TnC, which is capable of binding Ca^2+^; TnT, which binds to tropomyosin in muscle fibers maintaining the bond between actin and myosin; and TnI, which binds to actin in thin myofilaments in order to hold the Tn/tropomyosin complex together, inhibiting the binding of actin and myosin filaments [10,11,12]. The N-terminal region of cardiac TnT and the COOH-terminal region of TnI can undergo a proteolysis by μ-calpain [13,14]. An increase in calpain activity has been observed to cause alterations in myocardial contractility in certain cardiac diseases, while the administration of calpain inhibitors improved cardiac function [15,16]. HSP-27 is somehow involved in cardiac contraction, because ischemia and thermal shock induce a translocation of HSP-27 to the bands Z in cardiac tissue [17,18,19]. The stabilization of actin filaments occurs thanks to the different phosphorylation/dephosphorylation processes, which prevent the degradation and depolymerization induced by oxidative damage or thermal shock [20]. This stabilization seems to be an action caused by the non-phosphorylated form, which is responsible for the HSP activity as a molecular chaperone [21,22], and also participates in the solubilization of the aggregates of denatured proteins after stress [23].

Other functions for HSP-27 would be the modulation of the redox state and the inhibition of apoptotic cell death [24,25] which it is suggested to be mediated by the phosphorylated form [25]. The inhibition of cell death occurs through the direct interaction of HSP-27 with members of the apoptotic machinery that lead to the suppression of the activity of the enzyme caspase, one of the main actors responsible for cell death. A wide range of human diseases and the administration of some drugs have been associated with endoplasmic reticulum stress, which can lead to oxidative stress and therefore, can create an increase in reactive oxygen species (ROS). Endoplasmic reticulum damage induces an increased HSP-27 expression, preventing the generation of ROS and caspase-3 activity and avoiding cell death [26]. In addition, HSP-27 overexpression inhibits cytochrome C release and, consequently, the translocation of pro-death molecules to mitochondria [27].

It is important to highlight that HSP-27 is expressed in both constitutive and inducible form in the cardiac tissue, which gives it an important cardioprotective role. This cardioprotective effect has been extensively studied in animal models. In those studies, it was observed an increase in cell survival against lethal damage caused by cardiac ischemia in mice that overexpress HSP, compared to those that do not overexpress the protein [28]. It has been suggested that HSP-27 contributes to the maintenance of membrane integrity in different types of stress [29], protecting the heart through its functions as chaperone, facilitating the reconstitution of the cytoskeleton after stress. HSP-27 also works as an inducer of antioxidant mechanisms, since the production of free radicals is involved in the generation of myocardial lesions [30,31,32]. Thus, HSP-27 is considered as a potential target for the treatment of myocardial ischemia [12].

In conclusion, HSP-27 increased levels in different tissues suggest a protective role against different type of stress that cause cellular damage. HSP-27 could be considered as adaptive response proteins which enhance survival.

## 3. Stress/Addiction and Heart

It has been evidenced that there is a close relationship between stress and addiction. Exposure to both psychological and physiological stress at any stage of the addiction cycle seems to worsen this disease, increasing drug-seeking, drug-taking, and the number of relapses [33]. Stress is a common risk factor for development of both addiction and cardiovascular disease. Like stress, drugs of abuse lead to the activation of two systems: the catecholaminergic system and the hypothalamic-pituitary-adrenal (HPA) axis. They are considered anti-reward systems and play a fundamental role in the aversive effects, which are responsible for drug relapse, the main problem of addictive processes. Activation of both elements results in enhanced circulating catecholamine levels, which were shown to induce microfocal fibrosis that can damage the heart [34]. Numerous areas of the central nervous system (CNS) are involved in the integration between behavioral changes and the cardiovascular response associated with drugs of abuse. The presence of opioid and opioid receptors in the autonomic nuclei of the hypothalamus, which regulate the autonomous nervous system through projections to brain stem and spinal cord, is important for the regulation of cardiovascular function. Thus, the extrahypothalamic and hypothalamic stress systems contain corticotrophin releasing factor (CRF) neurons and receive noradrenergic innervation, which has been regarded to be critical for stress and addiction [35,36,37]. HPA axis regulates CRF transcription, adrenocorticotrophin hormone (ACTH)**,** and corticosterone secretion. This axis activates noradrenergic neurons in the nucleus of the tractus solitarius and the ventrolateral medulla, which are implicated in cardiac function [38,39]. These connections between the CNS and the heart support the idea that brain adaptive changes induced by drugs also affect to cardiac pathways, neurotransmitters, and different receptors (adrenergic, opioid of CRF) expressed in the heart. However, there is an intrinsic cardiac plexus, capable of functioning independently, that receives inferences from the sympathetic nervous system and parasympathetic nervous system. This intrinsic cardiac plexus regulates the response of intracardiac neurons and can send projections to different areas of the CNS mainly related to emotions. The fact that the heart receives projections from CNS and sends projections to the brain has made this organ regain great importance as an independent motor and driver of brain changes in certain pathologies [40]. The adaptive cardiac changes that occur after drug administration could be due to the activation of brain neuronal circuits and/or adaptive changes in intrinsic cardiac neurons, independent of the CNS. Supporting this hypothesis, previous studies [41,42] have shown that morphine withdrawal syndrome induced by naloxone methyl methiodide (quaternary derivative of naloxone that does not cross the blood–brain barrier) produces cardiac noradrenergic hyperactivity and increased c-Fos expression in different cardiac tissues, similarly to the cardiac changes observed after naloxone administration. These data suggest that some of the adaptive changes that occur during morphine withdrawal syndrome are due to peripheral mechanisms, independent of the CNS.

Nevertheless, it has been accepted that drugs of abuse induce considerable stress response, which brings the heart to adaptive changes that can lead to different types of cardiac arrhythmias consequence of an increased sympathetic activity. Thus, naloxone-induced withdrawal causes an increase in myocardial normetanephrine (extraneuronal noradrenaline metabolite) and noradrenaline (NA) turnover, with an increased activation of tyrosine hydroxylase (TH). The treatment with a CRF1 antagonist blocks these changes and demonstrates that CRF/CRF1 receptor activation is involved in the cardiac sympathetic activation seen after morphine withdrawal [43]. Heroin and other opioids can cause arrhythmias and non-cardiac pulmonary edema and may reduce cardiac output [44]. In addition, it has been postulated that heroin affects the sinus node and the surrounding nerves, ganglia, and atria myocardium, replacing their tissues with amounts of fatty and/or fibrous tissues. These changes may result in a dysfunction of the previously mentioned structures and, probably, to be the origin of different types of arrhythmias, which are partly responsible for the sudden cardiac death observed in heroin addicts [45]. Supporting this, ethanol and 3,4-methylenedioxymethamphetamine co-administration and methamphetamine increased NA turnover and TH expression and phosphorylation in the heart [46,47]. Not only chronic methamphetamine administration, but also withdrawal activates the brain CRF system associated with the heart sympathetic control and points towards a methamphetamine withdrawal-induced activation of sympathetic pathways in the heart [48]. Together with the enhancement of sympathetic activity, drugs of abuse and withdrawal induce an increased HSP-27 expression and phosphorylation. This enhancement suggests that this chaperone protects the heart against the cardiac changes observed after binge ethanol associated to methylenedioxymethamphetamine, methamphetamine and their withdrawals. Probably, an action that is made possible through HSP-27 antiapoptotic and oxidative stress-attenuating properties [46,47,48]. These investigations provide further insight into the specific biological mechanisms underlying the devastating cardiovascular consequences of drugs of abuse. Besides, they supply information about potential targets for the development of novel and effective pharmacotherapies to reduce cardiovascular- and stress-related complications associated with drugs of abuse.

## 4. Morphine Addiction and HSP-27

It is postulated that activation of µ-opioid receptor induces negative inotropic effects, together with an increase in the action potential duration. Both effects contribute to decrease myocardial infarction frequency [49,50,51,52]. However, there are very few studies about the effects of chronic activation of these receptors. It has been determined that chronic morphine exposure induces cardioprotective effects [53] and numerous neuroadaptive changes in cardiac noradrenergic systems. Thus, previous studies have detected that chronic activation of the µ-opioid receptor modifies sympathetic activity in the heart [54,55] and NA plasma levels [56,57]. In accordance with these studies, it has been demonstrated that chronic morphine treatment produces a decrease in baseline mean arterial blood pressure and heart rate. However, blocking the opioid receptor by naloxone in morphine addicted patients or in morphine-dependent animals unmasks the effects of chronic morphine administration resulting in increased sympathetic activity together with enhanced NA plasma concentrations [56], NA turnover [57] and TH expression [58]. Morphine withdrawal increases mean arterial blood pressure (MAP) and heart rate, but decreases NA levels in cardiac tissue, while normetanephrine and NA turnover are increased. This increase in the extraneuronal NA metabolite, generated by catechol-O-methyl transferase (COMT), occurs through an increase in the two isoforms of COMT: membrane (MB)-COMT and soluble (S)-COMT, suggesting that both isoforms of COMT are implicated in the degradation of NA [59]. Recently, it has been postulated that COMT has an important role inducing reward [60] and the activity of this enzyme has been linked to the vulnerability to develop addiction [61,62]. In addition, morphine withdrawal causes increased phosphorylation (activation) of TH at Ser40 and Ser31 suggesting that phosphorylation of TH may be an important modulator of TH activity during naloxone induced morphine withdrawal and it might be directly involved in regulating NA turnover in morphine withdrawn rats [43].

The toxic effects induced by opioids in vitro cause a severe stress situation that can trigger a neuronal degeneration accompanied by an increased metabolism of dopamine. This increase would be responsible for the production of oxygen free radicals, capable of causing oxidative stress [63]. Cells respond to this stress by increasing the synthesis of various cell defense proteins, HSP among them [64]. Similarly, it is widely known that opioid withdrawal causes a severe stress situation in the heart revealed by the increased metabolism of NA. This enhancement could induce oxygen free radicals that inhibit the mitochondrial respiratory chain activity in the cardiomyocites and subsequently, cell death. Despite all the damage inflicted to the cardiomyocites during morphine withdrawal, they still can increase HSP-27 expression and phosphorylation, which could protect the heart. Thus, morphine withdrawal syndrome increases HSP-27 expression and phosphorylation at Ser 15 and 82 [31,65]. The increase in the non-phosphorylated form of HSP-27 has a function as chaperone; therefore, the increase in HSP-27 expression would aim to repair proteins with a poor conformation and to prevent oxidative stress [2] that would have been generated during the drug-induced stress situation. Otherwise, HSP-27 phosphorylation leads to greater stability of the cytoskeleton, by inhibiting actin polymerization [66], which has anti-apoptotic properties, preventing cell death [67]. The activation (phosphorylation) of HSP-27 observed during withdrawal syndrome would enhance the protective function initiated by HSP during chronic morphine administration. HSP-27 is phosphorylated by the extracellular signal-regulated kinase at its residues of Ser 15, 78, and 82 [68,69]. Administration of [amino[(4-aminophenyl)thio]methylene]-2-(trifluoromethyl)benzeneacetonitrile (SL-327), a selective inhibitor of mitogen-activated extracellular protein kinase, decreases the HSP-27 phosphorylation at Ser 15 and 82 and the HPA axis activation observed during morphine withdrawal [59,65]. In addition, administration of propranolol, which reduces ACTH plasma levels, also inhibits HSP-27 phosphorylation during the morphine withdrawal syndrome, suggesting that β-adrenergic cardiac receptors blockade attenuates the cardioprotective role of HSP-27 [59]. In line with these results, the administration of alprenolol, another β-adrenergic blocker, has been shown to attenuate the cardioprotection induced during the ischemic preconditioning phase [31]. These results suggest that blocking β-adrenergic receptors in stressful situations can abolish the endogenous cardioprotective mechanisms that appear when baseline physiological conditions are altered. Besides, together with the increased ACTH plasma concentrations mentioned, during morphine withdrawal there is an increase in NA circulating levels [70] and in NA turnover in the heart [58]. This enhancement could support the hypothesis that peripheral catecholamines augmented levels would be an important mechanism involved in the excitatory effects produced by the precipitation of withdrawal syndrome on the HPA axis. The reduction in ACTH plasma levels, additionally with the decrease in HSP-27 phosphorylation, suggests that HSP-27 activation (phosphorylation) could be mediated by ACTH. In this sense, it has been shown that stress induces an increase in HSP-70 expression in adrenal capsules [64,71], which was abolished in hypophysectomized rats [71,72]. Furthermore, it has been observed a decrease in HSP-27 and HSP-70 expression in adrenocortical tumors with high levels of plasma cortisol, which lead to ACTH inhibition [73], suggesting a possible link between ACTH and HSP-27.

**Figure 2:**
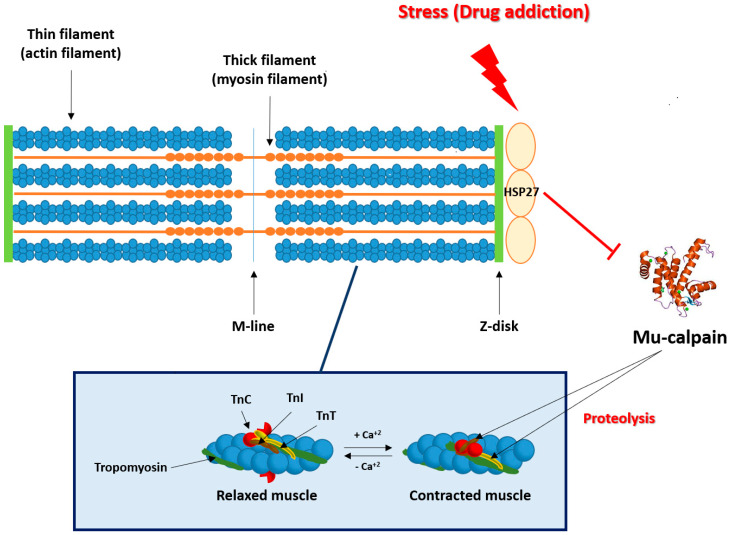
During stress situations, like drug addiction, HSP-27 functions as an excellent cardioprotective protein due to its properties as a chaperone and also in the stabilization of actin filaments. In these situations, HSP-27 is translocated to the Z bands in order to stabilize the actin myofilaments, protecting its degradation. During morphine withdrawal, HSP-27 could protect cardiac troponin T (cTnT) from µ-calpain degradation, whose expression is increased during withdrawal symptoms.

Alternatively, morphine withdrawal induces an important stressful situation, which in turn produces a destabilization of actin myofilament contributing to its degradation. HSP-27 stabilizes actin filaments producing a translocation of HSP-27 to the Z bands [18,74]. This translocation seems to be crucial for the stabilization of actin myofilaments, protecting its degradation [19]. During the morphine withdrawal syndrome, there is an increase in cardiac troponin T (cTnT) expression together with a decrease in cardiac troponin I (cTnI) expression, and a co-localization between cTnT and HSP-27 which has been described by immunofluorescence [75]. These results indicate that HSP-27 could protect cTnT from protease degradation and specifically from proteolysis by µ-calpain, whose expression is increased during withdrawal symptoms. It is known that µ-calpain produces a proteolysis of troponins to adjust muscle contractility to different stress conditions [76] (Figure 2). Troponins are responsible for the interaction of actin with myosin and, therefore, for cardiac contraction. If troponins are degraded by the increase in µ-calpain observed during morphine withdrawal, there will be a significant alteration of the contraction-relaxation mechanisms. Although the mechanism by which HSP-27 interacts with cTnT is not well established, all the results commented above support the hypothesis that HSP-27 avoided the degradation of cTnT by µ-calpain. HSP-27 also improved cardiac contractility during morphine withdrawal. Moreover, it has been demonstrated that activation of calpain increases cardiac injury during ischemia and reperfusion by sensitizing mitochondrial permeability transition pore. This sensitization results in opening and impairing mitochondrial metabolism through damage of complex I, while calpain inhibition decreases cardiac injury and improves heart function [77,78]. In addition, a recent study shows that post-treatment with the HSP-inducing compound geranylgeranyl acetone increases HSP-27 expression and enhances recovery from tachypacing-induced contractile dysfunction in cardiomyocytes [79].

Several studies have postulated an important role for CRF/CRF1 receptors in anxiety and in somatic, molecular and endocrine alterations evidenced during morphine withdrawal [80,81]. Despite the extensive research supporting the role of CRF in drug addiction, it is undetermined in drug-induced cardiac alterations. Provided that stress is a common risk factor for both addition and cardiovascular disorders, investigating the neuronal pathways or substrates that mediate the stress response may provide clues to understand the shared pathophysiology that links addiction and cardiac disorders. Although under normal physiological conditions, CRF1 receptors have only a scant presence in the heart [82,83,84], their levels are upregulated under cardiac disease [85].

Beyond CRF function as primary activators of the HSP system, CRF and its analogues also have effects on the heart and vasculature by increasing the secretion of NA and adrenaline from the sympathetic nervous system and adrenal medulla, respectively, and play a role within the heart as local mediators [86]. CRF1 receptor is necessary for the functioning of the chromaffin cells of the adrenal medulla. It has been shown that the destruction of this receptor leads to alterations in adrenaline biosynthesis [74]. In addition, administration of CRF1 receptor antagonists decreases the enhancement in plasma ACTH concentrations observed in rats with heart failure [87].

Regarding the role that CRF/CRF1 receptor pathway plays in the cardiac adaptive changes observed during naloxone-precipitated morphine withdrawal, several studies have shown that administration of CP-154,526 (CRF1 receptor antagonist) inhibited the increase of NA turnover and the phosphorylation of TH in cardiac tissue. CP-154,526 also attenuated the higher activity of hypothalamic-pituitary-adrenocortical axis (HPA), which is revealed by an enhancement of ACTH and corticosterone plasma levels [43]. Moreover, the increase in ACTH plasma and corticosterone levels, MB-COMT, S-COMT, NA turnover, and HSP-27 expression and phosphorylation observed after naloxone administration to morphine treated rats were significantly blocked in CRF1 receptor TR mice. Altogether, these data demonstrate that CRF/CRF1 receptor activation may contribute to stress-induced cardiovascular dysfunction after naloxone-precipitated morphine withdrawal. Finally, these results suggest that CRF/CRF1 receptor pathways could contribute to cardiovascular disease associated with opioid addiction, offering insight into potential therapy to treat this condition [88].

## 5. Conclusions and Future Perspective

In this review, we have focused mainly on the role of HSP-27 in the stressful situations that surround the addictive processes and, more specifically, in opioid addiction. Addiction is a complex process, where different intracellular messengers converge forming networks and interactions at multiple levels. Cardiac adaptive changes observed in addicted subjects would be the result of the coordinated actions of multiple cellular messengers that intervene, in turn, in multiple molecular pathways. Thus, current studies show an increased HPA axis and sympathetic pathways activity in cardiac tissues from morphine withdrawn mice. Nevertheless, addiction processes were able to increase HSP-27 expression and phosphorylation, which has cardiac protective effects. These proteins protect the heart, probably preventing cTnT degradation via reducing mu-calpain with cTnT interaction. In conclusion, HSP-27 represents a potential therapeutic target to address the adverse consequences of drug withdrawal, and further detailed studies on the HSP-27 may help to identify new targets to treat opioid addiction.

## Abbreviations

ACTH	adrenocorticotrophin hormone
CNS	central nervous system
COMT	catechol-O-methyl transferase
CRF	corticotrophin releasing factor
cTnI	cardiac troponin I
cTnT	cardiac troponin T
ERK	extracellular signal-regulated kinase
HPA	hypothalamic-pituitary adrenal
HSP	heat shock proteins
PKC	protein kinase C
ROS	reactive oxygen species
